# The myth of reproducibility: A review of event tracking evaluations on Twitter

**DOI:** 10.3389/fdata.2023.1067335

**Published:** 2023-04-05

**Authors:** Nicholas Mamo, Joel Azzopardi, Colin Layfield

**Affiliations:** ^1^Department of Artificial Intelligence, Faculty of Information and Communication Technology, University of Malta, Msida, Malta; ^2^Department of Computer Information Systems, Faculty of Information and Communication Technology, University of Malta, Msida, Malta

**Keywords:** Topic Detection and Tracking, event tracking, Twitter, evaluation methodologies, reproducibility, event modeling and mining

## Abstract

Event tracking literature based on Twitter does not have a state-of-the-art. What it does have is a plethora of manual evaluation methodologies and inventive automatic alternatives: incomparable and irreproducible studies incongruous with the idea of a state-of-the-art. Many researchers blame Twitter's data sharing policy for the lack of common datasets and a universal ground truth–for the lack of reproducibility–but many other issues stem from the conscious decisions of those same researchers. In this paper, we present the most comprehensive review yet on event tracking literature's evaluations on Twitter. We explore the challenges of manual experiments, the insufficiencies of automatic analyses and the misguided notions on reproducibility. Crucially, we discredit the widely-held belief that reusing tweet datasets could induce reproducibility. We reveal how tweet datasets self-sanitize over time; how spam and noise become unavailable at much higher rates than legitimate content, rendering downloaded datasets incomparable with the original. Nevertheless, we argue that Twitter's policy can be a hindrance without being an insurmountable barrier, and propose how the research community can make its evaluations more reproducible. A state-of-the-art remains attainable for event tracking research.

## 1. Introduction

The event tracking community has an evaluation challenge. The contemporary event tracking problem, formally known as Topic Detection and Tracking (TDT), has a straightforward task: to detect newsworthy events from tweets. The task should not require a more complex evaluation methodology than Information Retrieval (IR)'s: a labeled dataset and standard metrics. Twitter, however, forbids researchers from sharing full tweet datasets (Twitter, 2020a). Because Twitter does not let researchers share full tweet datasets, they cannot share a ground truth either, and because researchers cannot share a ground truth, they must annotate their algorithms' outputs manually. Event tracking has no reproducible evaluation methodology, no standard procedure to compare algorithms, and thus, no state-of-the-art.

It would be harsh to blame Twitter alone for the evaluation challenge. The difficulties to analyze event tracking algorithms also have roots in an older, more fundamental problem: defining the nature of events. Since the research area's inception, the event tracking community has not been able to agree on a common definition of events (McMinn et al., 2013; Farzindar and Khreich, 2015; Saeed et al., 2019a). The interpretations in Table 1 range diametrically from the theoretical to the practical and from the structural to the nebulous. A common ground truth of events cannot exist without a common definition of events either.

**Table 1:** Event tracking literature does not share a common definition of events, to the detriment of evaluation methodologies.

**Publication**	**Event definition**
Allan et al. (1998b)	“... something that happens at a particular time and place”
Mohd (2007)	“An event comprises at the very least what happened, where it happened, when it happened, and who was involved”
Panagiotou et al. (2016)	“In the context of online social networks, (significant) event *e* is something that causes (a large number of) actions in the OSN [Online Social Network]”
Chen and Li (2020)	“An action, or a series of actions, or a change that happens at [a] specific time due to specific reasons, with associated entities such as objects, humans, and locations”

The selected interpretations above describe events in different ways, from the more theoretical to the more practical. We refer to McMinn et al. (2013) and Saeed et al. (2019a) for more detailed discussions about different definitions of events.

Event tracking research has no solution to the evaluation challenge. What remains from more than a decade of efforts since Twitter's launch are an abundance of *ad hoc* evaluations (Weiler et al., 2017). Event tracking courts new applications in literature and in the news industry, where it can aid in newsgathering efforts (Beckett, 2019; Newman, 2022), but it courts them without a reliable measure of progress. Even worse, as we show throughout this review, misleading evaluations have filled the void of reproducibility. The evaluation challenge has become the evaluation problem. In this review, we investigate the evaluation problem through 79 publications as we make the following contributions:

- The task of evaluating event tracking algorithms remains a largely misunderstood problem, both on Twitter and elsewhere. In the absence of a common vision on how to evaluate algorithms, *ad hoc* studies have pervaded the research area (Weiler et al., 2017). In this paper, we present the most comprehensive survey yet on event tracking literature's evaluations on Twitter.
- Dataset reuse has consumed literature's idea of reproducibility, but on Twitter, the practice has no justification. To the best of our knowledge, the research community has never questioned how Twitter's data sharing policy affects datasets. In this paper, we demonstrate how tweet corpora tend to lose noise at a much higher rate than valid tweets, thus confirming that dataset reuse cannot stand for reproducibility.
- Our review portrays an unruly scene that Twitter's policies alone cannot excuse. Nevertheless, the rise of new applications for event tracking keep alive the call for reproducible research, measurable progress and a state-of-the-art. In this paper, we propose several ways how event tracking literature can make its data, ground truths, metrics and algorithms more reproducible.

The rest of this paper is structured as follows. In Section 2, we describe how we conducted this review. In Section 3, we discuss the virtues and flaws of manual evaluations before we shift our attention to automatic evaluations in Section 4. Then, in Section 5 we explore the issues of reproducibility in data, ground truths, metrics and algorithms, and in Section 6 we propose how each aspect could be made more reproducible. We summarize our findings in Section 7.

## 2. Review methodology

Our review covers 79 studies published between 2009 and 2022. We originally chose the publications for a non-systematic Ph.D. literature review about event tracking on tweets. A few of the studies also evaluate on other types of media in addition to tweets, but we always focus on the Twitter-based analyses for two reasons. First, Twitter's freely-available, easily-accessible data quickly established the social network as event tracking literature's preferred medium (Petrović et al., 2013). Second, Twitter's voluminous streams and data sharing policy (Twitter, 2020a) pose unique challenges that shape the research area's evaluation customs. While we focus on Twitter-based literature, many of our findings and proposals apply to the broader event tracking task, on Twitter and elsewhere.

In addition to event tracking research, our review also includes publications on event modeling and mining. The research area grew recently out of the need to manage, query and reason about events intelligently, which event modeling facilitates by representing events formally (Chen and Li, 2020). Often, modelers build on event extractors or trackers to discover events, and in this review, we consider how authors evaluated the event tracking components. We identified five broad categories of evaluation methodologies in our review:

- *None*: Publications that include no experiments, evaluating the quality of an algorithm neither quantitatively nor qualitatively.
- *Empirical*: Publications that include only a qualitative discussion without quantifying performance.
- *Manual*: Publications that include formal quantitative analyses based exclusively on human annotation.
- *Semi-automatic*: Publications that combine manual annotations with an automatic methodology. A semi-automatic analysis may include separate manual and automatic evaluations, or it may involve the manual annotation of a corpus which researchers later use to evaluate automatically.
- *Automatic*: Publications that eliminate manual input altogether, normally by using previously-published, annotated corpora.

We discuss manual evaluations in Section 3, and semi-automatic and automatic evaluations in Section 4. When we discuss automated evaluations, we further split methodologies into five categories:

- *Other*: Publications that follow original evaluation methodologies that the research community never widely-adopted.
- *Validity indices*: Publications that gauge the quality of clustering-based techniques using automated measures.
- *Keyword matching*: Publications that use a ground truth composed of a list of keywords, against which they compare an event tracker's outputs, themselves lists of keywords.
- *Window classification*: Publications that use an annotated corpus that has been segmented into time windows, each linked with zero or more events.
- *Document classification*: Publications that use an annotated corpus whose documents have been linked with zero or more events.

Following Weiler et al. (2019), in Sections 5, 6 we split reproducibility into four factors: the data, the ground truth, the metrics, and the algorithms. As part of our analysis on the data's reproducibility, we noted the number and sizes of the datasets used in each publication. The number of datasets lends credibility and generalizability to the findings, whereas dataset sizes reflect how scalable or sensitive an algorithm is. We only considered the number of datasets used in the specified event tracking task, which focuses on particular events; the unspecified event tracking task detects breaking news from general streams and seldom requires more than one dataset.

To analyze the reproducibility of the ground truth and the metrics, we noted who annotated the event tracking algorithm's outputs. Annotators influence reproducibility directly because they often interpret what it means for an event to be valid. Unfortunately, in many cases the authors failed to identify the annotators explicitly, which itself foreshadows the lack of reproducibility. We distinguish between two types of annotators in empirical, manual and semi-automatic evaluation methodologies, namely:

- *Researchers*: Publications that involve the studies' authors themselves evaluating the algorithms, including their own.
- *External*: Publications that employ students, Amazon Mechanical Turk workers or anyone else without authorship to evaluate the algorithms.

Finally, to analyze the reproducibility of the algorithms, we noted the baselines used in each publication. We distinguish between five types of baselines:

- *None*: Publications that only evaluate the quality of novel techniques and thus make no attempt to establish a state-of-the-art.
- *Parameter tweaking*: Publications that establish the novel techniques' optimal configurations but do not give any other context to performance.
- *Trivial algorithms*: Publications that invent new methods as benchmarks, generally confirming only that the novel techniques out-perform the simplest of algorithms.
- *Published algorithms*: Publications that give context to the quality of novel techniques by comparing them with other peer-reviewed solutions.
- *Published results*: Publications that compare the results of novel techniques with the peer-reviewed ones of other algorithms, lending objectivity to findings.

Due to space constraints, we provide the full list of 79 publications in this review, alongside our annotations, as Supplementary material. In the rest of this paper, we discuss the publications from different aspects: the flaws of manual evaluations, the futility of automatic alternatives and the matters of reproducibility.

## 3. Event tracking's manual evaluations

Event tracking literature knows well the challenges of evaluating its algorithms. Weiler et al. (2017), whose body of work (Weiler et al., 2015a,b, 2016, 2019) gives a broad overview of the problems, describe the evaluation process itself as “a challenging research question” independent of the event tracking one. Nevertheless, for a problem as essential as measuring progress, we still understand event tracking evaluations poorly. We still only seem aware that a problem exists, but we understand neither its cause nor its effects.

Evidently, we know that event tracking desperately needs a reproducible evaluation methodology (Weiler et al., 2019). We also know that manual evaluations, the very antithesis of reproducibility, abound; in Weiler et al. (2017)'s review, 18 of 42 publications evaluated manually. Only publications with empirical analyses or no analyses at all outnumbered manual evaluations–19 of 42. Still, the full implications of Weiler et al. (2017)'s review–the implications of a research area without an established evaluation process, without a state-of-the-art–continue to escape event tracking literature. In this review, we broaden our understanding of the evaluation problem on Twitter data.

We start with a review of manual evaluation methodologies. Our findings largely confirm (Weiler et al., 2017)'s: manual evaluations continue to dominate the landscape of event tracking analyses. 38 of 79 studies (48.10%) in our review depend entirely on manual annotations. Fully-automated and partially-automated analyses constituted less than a third: 25 studies (31.65%). Of the rest, we could only glean the evaluation process of seven (8.86%), which evaluated empirically or not at all. There is not much to say of the last seven except that they do not measure any form of progress and run contrary to the principle of reproducibility. Therefore in the rest of this section, we focus on the challenges of manual evaluations: the manual costs and their ramifications, and the inherent subjectivity that they all bear.

### 3.1. Manual costs

Manual evaluations have always drawn criticism for their costs. Chen et al. (2013) lamented the difficulties “to obtain [the] human relevance judgment[s]” necessary for a manual evaluation, and more colorful descriptions tell of a “daunting” process (Meladianos et al., 2015), a time-consuming ordeal that implies “an overwhelming amount of effort” and renders manual evaluations “infeasible in practice” (Aiello et al., 2013). The manual efforts make themselves manifest.

Far from being infeasible, manual evaluations remain ubiquitous in research. Apart from 38 manual analyses (48.10%), researchers in 14 semi-automatic evaluations (17.72%) mixed manual evaluations with automatic ones or created tailored ground truths manually for use in automatic evaluations. Even Meladianos et al. (2015), who labeled the process “daunting,” ultimately repeated the same manual process to annotate the ground truth in a follow-up study (Meladianos et al., 2018). Event tracking literature keeps finding itself inexplicably lured back to manual evaluations.

Evidently, the challenges persist despite the prevalence of manual evaluations. They remain plagued by the problems that Aiello et al. (2013), Chen et al. (2013), and Meladianos et al. (2015) deplored. Manual evaluations still require considerable human and financial efforts (Farzindar and Khreich, 2015; Saeed et al., 2019a) to construct the ground truth or to annotate the algorithms' outputs. The challenges persist with a forceful intensity; financial costs compelled researchers from 19 of 52 studies with a manual component (36.54%) to annotate the outputs themselves, and Gu et al. (2011) shared the burden with an external reviewer. Manual evaluations are not and cannot be scalable (Weiler et al., 2015a).

### 3.2. Ramifications

Event tracking literature's protests cease at the obvious, the efforts of manual evaluations, but the problems do not. The same efforts cause other, less cited ramifications. Consider what manual evaluations measure. Event tracking research forks into two: document-pivot techniques cluster tweets to form events, and feature-pivot techniques find events in changing features, like a burst in volume or a shift in discourse. When evaluating, however, the human annotator must somehow determine what story a cluster tells or what caused a feature to change. Human evaluations require human-understandable representations of events.

Literature describes events in different ways. In document-pivot techniques, literature describes events with the tweets closest to the cluster's centroid (Akhtar and Siddique, 2017; Liu et al., 2017), a rudimentary form of summarization. In feature-pivot techniques, the solution comes more laboriously. Some methods identify only a burst in tweeting activity: an event but not its cause (Buntain et al., 2016; Hsu et al., 2018). Others identify a set of keywords to form a narrative (Hsieh et al., 2012), but scattered keywords tell an interpretive story (Lanagan and Smeaton, 2011; Aiello et al., 2013; Hasan et al., 2019).

In short, what event tracking literature purports to measure differs from what it actually measures. Research does not measure the central aspect of event tracking algorithms, how accurately they detect and track events. Research measures implicitly how well summarization algorithms describe, even while describing remains a secondary aspect of modern solutions. Perhaps no other evaluation typifies this disconnect better than Meladianos et al. (2015, 2018)'s, which evaluates algorithms on the outputs of a summarization component detached entirely from the event tracking component.

Manual evaluations have other ramifications too. Even non-empirical analyses often involve a heavy measure of empiricism. Research normally uses few baselines; every additional baseline symbolizes an additional output to annotate manually. And in those few baselines, empirically-set configurations reign; every additional configuration too symbolizes an additional output to annotate manually.

The alternative lies in evaluations such as George et al. (2021)'s. George et al. (2021)'s evaluation only includes two baselines, but the lengthy experiments to tweak the parameter-laden algorithms–including the authors' own–constrained the evaluation to a small sample of the data. Sacrifices like George et al. (2021)'s appear commonly. The 20 manual evaluations that provided dataset statistics in our review averaged just 5.95 corpora. Automated or partially-automated evaluations, which reduce the manual efforts, afforded more or larger datasets: an average of 6.75 corpora in 8 semi-automatic evaluations and 13.00 in 6 automatic analyses.

Such empiricism further erodes the reliability of evaluations. The costs of manual evaluations lead researchers to tread the delicate balance between thoroughness and generalizability. Failing to thoroughly-exhaust the parameter space can lead to sub-optimal configurations and misleading improvements, as Keogh et al. (2004) demonstrated elsewhere in IR research. Similarly, failing to evaluate on diverse or sufficiently-large datasets prohibits research from drawing generalizable conclusions about an algorithm's progress (McMinn et al., 2013).

### 3.3. Human error and bias

Of all ramifications of manual evaluations, none compare to human error and bias. Event tracking avoids the mere mention of subjectivity; only Weiler et al. (2015a), to the best of our knowledge, explicitly address human error and bias, and only to hurriedly evoke that it “might” exist. Of course subjectivity exists. Errors and bias seem inseparable from human participation and individualism. If event tracking research cannot agree on what constitutes an event (McMinn et al., 2013), how could we expect annotators to agree on what constitutes a valid event?

Look closely in event tracking literature, and you will find explicit examples of subjectivity. Swan and Jensen (2000) and Allan et al. (2002)'s shared experiment recruited four students to annotate groups of event-related features: keywords and named entities. The four students agreed so rarely (*kappa* = 0.233) on the annotations that the authors themselves refused to draw any conclusions. Few others measured inter-annotator agreement and only ever reached moderate levels (Mele et al., 2019; Pradhan et al., 2019). Look closer in event tracking literature, and you will find many more implicit examples.

While human error and bias do not surprise us, the lack of effort to minimize them does. Few attempted to annotate outputs systematically. Chakrabarti and Punera (2011) devised five labels to describe the state of American football games, plays within those games, and other general comments. Zhou et al. (2015, 2017) presented slightly more rigid rules based on Who does What, Where and When, but such examples appear scarcely. Most researchers leave the labeling process to the discretion of the annotators, and in discretion, subjectivity prevails.

Evidently, coming up with rules to minimize subjectivity proves challenging. Even ignoring the lack of a common definition of events (McMinn et al., 2013; Farzindar and Khreich, 2015), whatever understanding of newsworthiness we adapt, some events tread the fine line between newsworthy and trivial. Hsieh et al. (2012)'s rules, in particular, typify the futile efforts to standardize manual annotations without eliminating human interpretation from the process. In Hsieh et al. (2012)'s guidelines, an event comprises either a set of popular tweets or a group of keywords that recount a coherent narrative.

Of the 79 publications in our review, only one stands out in the matter of human error and bias. The SNOW 2014 Data Challenge gathered groups of researchers in an event tracking contest, and it contains what may be the most reproducible of manual evaluations (Papadopoulos et al., 2014). The competition stands out for the clear way in which the organizers standardized the datasets, clarified different aspects of events and outlined the annotators' guidelines. It stands out for another reason too, however: the unguarded descriptions of the difficulties to design such a protocol. Papadopoulos et al. (2014) described the process as “highly complicated” and, similarly to Weiler et al. (2017), concluded that “properly assessing the performance of different methods constitutes a significant challenge on its own.”

Manual evaluations have thus come to an impasse. They remain with too few comparisons and too little data to reveal the qualities of algorithms and establish a state-of-the-art. A few innovated. Some researchers designed automatic evaluation methodologies to eliminate the manual efforts, the rampant empiricism, and human error and bias from the process. Still, none of the automatic methodologies succeeded in replicating faithfully the ideal evaluations of IR research, as we discuss next.

## 4. The futility of automatic evaluations

The first publications from the TDT pilot study (Allan et al., 1998a) depict the ideal event tracking evaluation. The ideal evaluation receives a corpus of documents, each with an unambiguous label: precise or imprecise; about one event or the other, or about no event at all. An automatic function processes an algorithm's output within seconds, not minutes or hours, and expresses the results in IR's well-defined metrics: precision and recall. The ideal evaluation thus requires minimal human intervention and effort, and since the majority of event tracking researchers follow the same process–the same dataset, ground truth and metrics–they do not need to implement a baseline. They only need to compare the results with those published elsewhere. With the ideal evaluation, event tracking has one undisputed state-of-the-art.

The ideal event tracking evaluation seems irrevocably reserved to the TDT pilot study and its immediate legacy. Twitter made labeling massive tweet corpora infeasible (Chen et al., 2013; Farzindar and Khreich, 2015) and restricted data sharing to stripped tweet IDs (Twitter, 2020a). The same labeling process would have to be repeated for every single study. Without a shared dataset and ground truth, the TDT pilot study's evaluation seems more quixotic than ideal.

Event tracking literature improvised. The manual efforts demanded the convenience of an automatic solution, but convenience could also bring the research area closer to the ideal evaluation. We split the resulting attempts into two groups: semi-automatic and automatic analyses. In semi-automatic evaluations, researchers normally annotate a ground truth manually in such a way that the algorithms' outputs can be evaluated automatically. In automatic evaluations, researchers generally reuse previously-labeled data to eliminate the manual component.

Table 2 lists all semi-automatic and automatic publications in our review. The 25 studies (31.65%) epitomize the research area's broader difficulties to evaluate: scattered solutions without a common approach. Some use validity indices, rough indicators of quality. Others adapt traditional classification to Twitter's massive corpora or measure performance by matching an algorithm's keywords to a list of ground truth keywords. In particular, in the rest of this section we focus on classification and keyword matching analyses, paragons of literature's ingenuity.

**Table 2:** A summary of semi-automatic and automatic TDT evaluation methodologies on Twitter.

**Publication**	**Evaluation**	**Type**	**Annotators**
Choudhury and Breslin (2011)	Semi-automatic	Document classification	External
Popescu et al. (2011)	Semi-automatic	Window classification	
Petrović et al. (2012)	Semi-automatic	Document classification	External
van Oorschot et al. (2012)	Automatic	Window classification	
Aiello et al. (2013)	Semi-automatic	Keyword matching	Researchers
Shen et al. (2013)	Semi-automatic	Window classification	External
Chierichetti et al. (2014)	Automatic	Window classification	
Corney et al. (2014)	Semi-automatic	Keyword matching	Researchers
De Boom et al. (2015)	Semi-automatic	Document classification	Researchers
Meladianos et al. (2015)	Semi-automatic	Window classification	External
Liu et al. (2016)	Semi-automatic	Other	
Preoţiuc-Pietro et al. (2016)	Semi-automatic	Document classification	External
Weiler et al. (2016)	Automatic	Other	
Edouard et al. (2017)	Automatic	Document classification	
Li et al. (2017)	Semi-automatic	Document classification	External
Huang et al. (2018)	Semi-automatic	Window classification	External
Meladianos et al. (2018)	Semi-automatic	Window classification	External
Choi and Park (2019)	Automatic	Keyword matching	
Saeed et al. (2019b)	Automatic	Keyword matching	
Weiler et al. (2019)	Automatic	Document classification	
Farnaghi et al. (2020)	Automatic	Validity indices	
Hettiarachchi et al. (2021)	Semi-automatic	Keyword matching	Researchers
Zhang et al. (2021)	Automatic	Keyword matching	
Di Corso et al. (2022)	Automatic	Validity indices	
Kolajo et al. (2022)	Automatic	Document classification	

Data is only filled-in for publications with clearly-described evaluation methodologies.

### 4.1. Document and window classification

A few logically let themselves be inspired by event tracking's early evaluations. Traditional classification, with labeled tweet corpora, remains prohibitively expensive, more so than the annotations of manual analyses (Petrović et al., 2010; Unankard et al., 2015; Weiler et al., 2017). Instead, early solutions labeled small subsets of full corpora. Petrović et al. (2010) annotated less than 0.01% of 50 million tweets, and McMinn et al. (2013), dissatisfied by Petrović et al. (2010)'s lean corpus, hired Amazon Mechanical Turk workers. Even then, McMinn et al. (2013) only labeled a small portion of a 120 million-tweet corpus: 152,950 tweets, or 0.13%.

Nearing–let alone matching–McMinn et al. (2013)'s accomplishments appears as a daunting prospect. Even if we had to ignore the issues of dataset re-usability that we describe in Section 5, tweeting behaviors change (Meladianos et al., 2015) and so do Twitter's features. It appears utterly infeasible and nonsensical to assemble a new dataset for every new set of requirements. Some researchers gave a new twist to traditional roots. If annotators could not label thousands of tweets, they could, at least, label time windows. Seven of 25 automated studies (28.00%) in our review classified time windows as eventful or not, or aligned timelines with a ground truth, itself a minute-by-minute timeline of events, usually from a news outlet. A precise event is simply one that co-occurs with a ground truth event.

Nonetheless, beneath the solution's elegant veneer lie troubling assumptions. Classification assumes that the ground truth is complete: that it captures every newsworthy occurrence, every general observation and every interesting statistic. It assumes that only one event happens at a time (van Oorschot et al., 2012), and that the algorithm and the ground truth capture it simultaneously (Meladianos et al., 2015, 2018). Finally, it assumes that the event tracking algorithm and the ground truth report events once and only once. The hopeful assumptions fail often in practice; during football matches, The Guardian commonly announces goals in blurbs and defers the details to a few minutes later, and the BBC mixes punditry with reporting. When the assumptions fail, the errors proliferate (van Oorschot et al., 2012).

In fact, semi-automatic classification achieves little in the way of reproducibility. It still depends on an annotator to manually project the algorithm's events onto the ground truth, lest the two should be misaligned (Shen et al., 2013; Huang et al., 2018). Moreover, when the events that Twitter finds interesting differ from what journalists find newsworthy (Marcus et al., 2011), then a human annotator may have to adapt the ground truth (Shen et al., 2013; Huang et al., 2018). Even when the assumptions hold, classification relapses to the error-prone and subjective ways of manual evaluations.

If we could solve the above problems, classification would still have a narrow scope. Classification expects of Twitter users the same behavior as the news media; a tweet must either describe an event or avoid it altogether. It forces spam and noise, opinions and redundant topics, and other difficult-to-enumerate events, like statistics and observations, to share one label: imprecise. Every event must fit in a rigid two-by-two confusion matrix: precise or imprecise, recalled or missed. Some researchers filter events manually to fit neatly in the matrix (Shen et al., 2013; Huang et al., 2018). Most acquiesce to the limits.

To summarize, classification's flaws lie in what it measures. Classification measures an event tracking algorithm's ability to detect events but not what it detects. An algorithm must detect and track, but in the pursuit of an automatic evaluation methodology, the research community largely abandoned the secondary role, to describe (Panagiotou et al., 2016). Classification says nothing about the quality of a document-pivot approach's clusters, nor about a feature-pivot approach's keywords. Keyword matching analyses partially overcame this issue.

### 4.2. Keyword matching

Describing events, not to mention evaluating the descriptions, represents a complex problem. Since Panagiotou et al. (2016) advocated for event tracking algorithms capable of describing events, the task has developed into an independent research area: event modeling and mining (Chen and Li, 2020). Describing events seems like a utopian standard for event tracking to uphold, but it should still make us question what our automatic evaluations measure. Keyword matching analyses do not demand event modeling's formal descriptions but simultaneously capture an algorithm's ability to detect and describe.

Keyword matching analyses stem from Hsieh et al. (2012)'s intuition: a few keywords can tell a story. That same year, Lee et al. (2012) conceived the idea of measuring how many of those keywords an event tracking algorithm could extract. The following year, Aiello et al. (2013) shared a dataset with keywords as the ground truth and popularized (Lee et al., 2012)'s methodology. In the years since then, many have reused (Aiello et al., 2013)'s corpus (Adedoyin-Olowe et al., 2016; Choi and Park, 2019; Saeed et al., 2019b). Several others replicated the process (Corney et al., 2014; Hettiarachchi et al., 2021; Zhang et al., 2021).

Notwithstanding their prevalence, keyword matching evaluations remain weak imitations of event tracking's ideal evaluation. The relatively-small sets of ground truth keywords do not cater to lexical or stylistic variety, and like in manual evaluations, researchers rarely follow a system to create the ground truth. In private correspondence, Aiello et al. (2013) described to us their process, how they chose the keywords manually themselves and had a journalist act as an editor. Hettiarachchi et al. (2021)'s process excluded the journalist altogether. In other words, keyword analyses still submit to the human error and bias of manual evaluations that automation should have eliminated.

Keyword matching evaluations falter at other challenges too. They exclude document-pivot and embedding-based approaches, which must adapt their outputs–clusters of tweets and abstract semantic dimensions–into human-readable keywords. Furthermore, like classification, keyword matching evaluations lump events and their keywords into one of two categories: precise or imprecise, recalled or missed. In short, automatic evaluations sacrifice reliability in the name of convenience, accuracy and objectivity.

Stuck between manual and automatic evaluations, event tracking literature's conundrum is not new. The challenges to find an automatic evaluation that solves the problems of a manual evaluation evoke summarization literature's own struggles. In summarization too, replicating human scrutiny in automatic evaluations proves difficult, and in summarization too, automatic methodologies fail to replace manual alternatives (El-Kassas et al., 2021). Summarization literature has not found a solution yet. It did, however, find a way for manual and automatic evaluations to complement each other.

Automatic evaluations should not threaten the existence of manual evaluations; the two can co-exist. Summarization literature's automatic methodologies, namely ROUGE and BLEU, measure content coverage; the manual ones capture the more human elements of a summary: comprehensiveness, clarity and objectivity (El-Kassas et al., 2021). Event tracking research too should accept the flaws of manual and automatic evaluation methodologies and endeavor to strengthen them, make them more reproducible. In the next section, we explore the issues of reproducibility in event tracking research's data, ground truths, metrics and algorithms.

## 5. Issues of reproducibility

The issues of reproducibility became obvious as we prepared this review. We struggled to identify how authors collected datasets and how they assembled the ground truth, and to grasp who annotated the output and on what criteria. We struggled to draw conclusions about how one algorithm compares with its baselines, and to align evaluation methodologies. In the rest of this section, we discuss these struggles. We develop and extend (Weiler et al., 2019)'s previous work to understand what deprives event tracking literature of reproducible data, ground truths, metrics and algorithms.

### 5.1. Data

Every problem of reproducibility has roots in Twitter's policy. Twitter only allows sharing tweet IDs (Twitter, 2020a), skeletal corpora needing to be downloaded anew. If the social network did permit data sharing, no problem would seem too formidable; after all, McMinn et al. (2013) already demonstrated that we could crowd-source annotations for massive datasets. Evidently, reusing datasets would not always be possible; a study may have its own requirements of the data (McMinn et al., 2013), and tweeting habits change over time: more noise, new features and longer tweets (Meladianos et al., 2015, 2018). At least, however, the possibility would exist.

A few endured Twitter's restrictions to explore the possibility. Petrović et al. (2010) shared a compliant dataset with labeled tweets from 27 events, McMinn et al. (2013) shared one with 152,950 tweets from 506 events, and Aiello et al. (2013)'s dataset spurred keyword matching analyses. The SNOW 2014 Data Challenge's organizers cleverly sidestepped Twitter's restrictions: instead of datasets, Papadopoulos et al. (2014) provided instructions to participants on how to collect datasets. Aside from the latter, however, the shared datasets give only a false sense of reproducibility. The first warning lies in the amount of lost data.

Crow (2020) called it dataset “rot.” Users might voluntarily make their accounts private or delete tweets, and Twitter routinely removes users who violate its rules (Twitter, 2020b), and with them, their tweets. Weiler et al. (2017) spent a week downloading a sample of tweets from McMinn et al. (2013)'s dataset, and they could only retrieve 40% of the sample. Hettiarachchi et al. (2021) retrieved just 65.80% of the whole corpus and Kolajo et al. (2022) 54.21% of all labeled tweets. Hasan et al. (2019) found old corpora to have rotted away beyond usability.

Many others found the same corpora to be perfectly usable. By all measures, the event tracking community adopted the scarce open-source datasets rapaciously. While the scale of missing tweets undermines reproducibility (Weiler et al., 2017), we might find it in ourselves to forgive the loss as long as the downloaded tweets followed an identical distribution as the original dataset. In other words, we could forgive dataset rot if only the unavailable tweets had been sampled randomly from the original dataset–the same corpus on a smaller scale. Dataset reuse in event tracking literature hinges on this assumption, which, to the best of our knowledge, research has never challenged before. The assumption, unfortunately, has no basis in reality.

To test the assumption, we re-downloaded four leftover datasets from our previous projects. We had collected the datasets shown in Table 3 between 3 years and 1 day earlier by tracking the event hashtags, and the names of the stadium, teams, coaches and players. Not more than 3 years had passed since we first downloaded the four datasets, but we had already lost between 12.61 and 35.78% of tweets. More worryingly, as Tables 4, 5 show, the data distribution had changed.

**Table 3:** Statistics about the original datasets, and the same datasets downloaded anew after a period of time.

	**Download date**		**Tweets**		**% Available**
	**Original**	**Downloaded**	**Original**	**Downloaded**	
Crystal Palace-Chelsea	Dec 30, 2018	Aug 29, 2021	63,891	41,028	64.22
Southampton-Arsenal	Jun 25, 2020	Aug 29, 2021	97,874	70,656	72.19
Turkey-Italy	Jun 11, 2021	Aug 30, 2021	109,888	90,543	82.40
Liverpool-Atlético de Madrid	Nov 3, 2021	Nov 4, 2021	107,607	94,040	87.39

The downloaded datasets are inevitably smaller than the original datasets due to some tweets becoming irretrievable.

**Table 4:** The change in mean values of selected attributes between the sets of unavailable and available tweets.

	**Change between unavailable and available tweets**			
	**Crystal Palace Chelsea**	**Southampton Arsenal**	**Turkey Italy**	**Liverpool Atlético de Madrid**
Average account age	28.62%	38.48%	46.49%	0.77%
Average number of followers	420.83%	338.50%	30.97%	391.41%
URLs per tweet	–22.85%	–26.29%	–57.62%	–69.80%
Mentions per tweet	–6.78%	–12.00%	–21.16%	–29.80%

Positive values mean that the value was higher in the available tweets than in the unavailable tweets, and vice-versa. For example, in the match between Turkey and Italy, the average account was 46.49% older for available tweets than for unavailable tweets.

**Table 5:** The percentage of available tweets calculated for selected groups with particular attributes.

	**Percentage of available tweets**			
	**Crystal Palace Chelsea**	**Southampton Arsenal**	**Turkey Italy**	**Liverpool Atlético de Madrid**
All tweets	64.22%	72.19%	82.40%	87.39%
Tweets by verified authors	91.59%	93.28%	91.54%	95.62%
Tweets by new accounts (age < week)	42.55%	48.37%	57.73%	69.49%
Tweets by authors without description	56.23%	68.42%	73.77%	88.85%
Tweets by authors without followers	48.95%	36.30%	38.19%	29.03%
Retweets	59.89%	66.71%	78.93%	79.66%
Tweets containing URLs	60.24%	67.87%	71.47%	68.57%
Tweets mentioning *stream*	7.33%	11.71%	9.05%	18.02%

For the downloaded dataset to be representative of the original dataset, the percentage of available tweets in each group should be approximately equal to the percentage of all tweets that were still available. Many meaningful metrics change drastically.

The average tweet changed. The percentage of available tweets dwindled steadily, from 87.39% 1 day after the match between Liverpool and Atlético de Madrid to 64.25% in the match between Crystal Palace and Chelsea 3 years earlier. The average tweet in the downloaded datasets contained 17.44% fewer mentions and 44.14% fewer URLs. In every match, retweets were more likely to have been deleted than the average tweet, and so were tweets with URLs. Within 1 day, more than 80% of tweets containing the word *stream* had already become unavailable, and the number rose further.

The average author changed too. Excluding the latest match, the average author of available tweets was between 25 and 50% older than the authors of missing tweets. Far fewer authors had empty profile descriptions; far more were popular. In the match between Crystal Palace and Chelsea, the authors of retrievable tweets averaged five times as many followers as those of irretrievable tweets. Only authoritative users seemed immune to change. Even when we lost more than a third of tweets, we retrieved more than 90% of tweets by verified authors.

The failing assumption disrupted even the temporal distribution. We lost tweets published early in a match disproportionately more than tweets published late. Only 69.45% of tweets published in the first 15 minutes remained available, as opposed to 81.27% of tweets published in the last 15 minutes. The number of available tweets rose and fell almost perfectly-inversely to the frequency of the word *stream* (Pearson correlation coefficient: *r* = −0.9622).

Finally, the changes happened quickly. The dataset from the match between Liverpool and Atlético de Madrid had morphed into an almost-unrecognizable event within 24 hours. By then, we had only lost 12.61% of tweets, but the ones that remained had noticeably far fewer mentions and far fewer URLs. The remaining authors had become older, more popular and more authoritative. It feels as if tweet datasets cease to be reusable the moment we collect them.

The changes are neither incidental nor entirely new. The average unavailable tweet resembles the tweets that event tracking researchers filter: harmful tweets, spam and noise. Over time, tweet datasets self-sanitize and become what the filters aspire to make them. Data does not decay uniformly but changes fundamentally. What Crow (2020) called dataset rot, we call dataset corruption.

We observed similar changes in other tweet datasets aside from our own. Waseem and Hovy (2016) and Founta et al. (2018) annotated two datasets to characterize spam and various forms of abuse. We downloaded the datasets in Table 6 between 4 and 8 years later, and retrieved two-thirds of normal tweets, as shown in Tables 7, 8. Of the rest, hateful and abusive tweets, far fewer remained. Only a third of Founta et al. (2018)'s abusive tweets remained, and almost no racist tweet in Waseem and Hovy (2016)'s dataset survived the purge. Only one class deviated from the trend; 80.25% of Waseem and Hovy (2016)'s sexist tweets remained available but only because the annotators disagreed on what constituted sexism and judged too harshly innocent tweets. Waseem and Hovy (2016)'s and Founta et al. (2018)'s datasets too self-sanitized.

**Table 6:** Statistics about the original datasets collected by Waseem and Hovy (2016) and Founta et al. (2018), and the same datasets downloaded anew after a period of time.

	**Download date**		**Tweets**		**% Available**
	**Original**	**Downloaded**	**Original**	**Downloaded**	
Waseem and Hovy (2016)	Apr 2013-Jul 2015	Oct 29, 2021	16,907	10,365	61.31
Founta et al. (2018)	Mar 2017-Apr 2017	Oct 28, 2021	99,799	53,641	53.75

The downloaded datasets are inevitably smaller than the original datasets due to some tweets becoming irretrievable.

**Table 7:** In Waseem and Hovy (2016)'s hate speech detection dataset, almost no racist tweet remained available.

**Percentage of available tweets (Waseem and Hovy**, **2016****)**	
All tweets	61.31%
Normal tweets	66.11%
Racist tweets	0.61%
Sexist tweets	80.25%

While most sexist tweets remained retrievable, the authors and the annotator could not agree on a labeling procedure, and in the end, few had a sexist element to them.

**Table 8:** In Founta et al. (2018)'s abuse detection dataset, tweets labeled as abusive, hateful or spam became unavailable at higher rates than normal tweets.

**Percentage of available tweets (Founta et al.**, **2018****)**	
All tweets	53.75%
Normal tweets	64.10%
Abusive tweets	34.35%
Hateful tweets	41.92%
Spam tweets	55.61%

Our findings have important ramifications on the practice of dataset reuse. Researchers reuse tweet datasets in vain attempts to establish a state-of-the-art, but how could they when datasets change so fundamentally? Kolajo et al. (2022) still used McMinn et al. (2013)'s dataset in 2022, and in 2019, Choi and Park (2019) and Saeed et al. (2019b) still used some version of Aiello et al. (2013)'s dataset from 2012. Neither needed to concern themselves with the precision-recall trade-off to the same extent as Aiello et al. (2013). In the end, how much of the improvements did they owe to algorithmic design? How much to the sanitized data?

Apparently-unaware of how tweet datasets self-sanitize over time, Weiler et al. (2019) proposed an alternative, the artificial stream. The artificial stream replaces the traditional corpus with a statistical distribution of background topics, formed by words, into which the researcher injects events, themselves formed by event-related keywords. In Weiler et al. (2019)'s vision, the artificial stream would simultaneously solve the issues of dataset reusability and automate the evaluation.

Nevertheless, replacing traditional corpora with synthetic ones seems reckless. The artificial stream only solves a narrow facet of the data problem. Like keyword matching evaluations, the artificial stream excludes document-pivot or embedding-based approaches. It misses the spontaneity and nuances of Twitter's discourse, like the prolonged, heightened discussion that follows extraordinary events (Lanagan and Smeaton, 2011). Moreover, the artificial stream only shifts subjectivity from the annotation to the dataset. The researcher decides the nature of the event: whether to simulate a quiet setting or a noisy one, and whether to adapt the data to the algorithm or pose it as a challenge.

The findings from our brief experiment rewind the state of event tracking evaluations to 2012. Back then, fresh from the struggle of annotating one of the first tweet datasets for event tracking research (Petrović et al., 2010, 2012) bemoaned “the lack of a corpus that could be used to measure performance.” When Petrović et al. (2012) wrote those words, the promise of a shareable tweet dataset remained a possibility, if a remote one. Now, a solution seems more complex, less definite, but perhaps not inconceivable either. We suggest ways to make event tracking's data more reproducible in Section 6.1.

### 5.2. Ground truth

Unlike the data, the ground truth never challenged event tracking literature. As a research area concerned with the newsworthy, event tracking could always rely on the news media for a reliable ground truth. In certain domains, fixed rules and clear boundaries even allowed event tracking research to construct ground truths almost effortlessly, from easily-enumerable events that leave no doubt about their veracity. In football matches, research tracks goals and yellow cards, and in American football, it tracks touchdowns.

Any issues of reproducibility do not arise from the ground truth itself but from the interpretation of it. Some events either happen or they do not: a player either scores a goal or they do not, either receives a yellow card or they do not. Other events, however, solicit interpretation. An injury, an offside and a clear goalscoring opportunity all weave the narrative of an event, but their place in the ground truth depends on the annotator's subjective judgment (Meladianos et al., 2015, 2018).

While a certain degree of interpretation seems unavoidable, researchers often take excess license. Shen et al. (2013) and Huang et al. (2018) filtered unpopular events, but should an event tracking algorithm not aspire to capture those too? Others filtered the ground truth of even the most easily-enumerable of events; Aiello et al. (2013) retained only “some key bookings” in football matches, and Nichols et al. (2012) and Meladianos et al. (2015, 2018) inexplicably ignored the start of the second half, despite including the start and end of the game, and half-time. A few annotated “significant” (Aiello et al., 2013) or “major events” (Marcus et al., 2011), but neither explained what made an event significant or major.

Subjectivity stems from a deeper place. Primarily, annotators must exercise their subjective judgment because no objective definition of events exists. The event tracking community has repeatedly attempted to define events, from the research area's first definition–“some unique thing that happens at some point in time” (Allan et al., 1998a)–to more contemporary ones that consider how events affect a subset of a social network (Saeed et al., 2019a). It could not, however, adopt a common definition (McMinn et al., 2013). The research community could only agree that events have valuable temporal and spatial dimensions, and a certain significance that, like Marcus et al. (2011)'s or Aiello et al. (2013)'s filtering, proves indescribable, and thus subjective (McMinn et al., 2013).

The differing definitions of events and the capricious interpretation of the ground truth undermine reproducibility. They inject subjectivity into a set of events that should be the pinnacle of objectivity. Few aspects of reproducibility could–or should–be as robust as the ground truth, but with its practices, event tracking literature turns it into a weak imitation of manual annotation. We suggest ways to make event tracking's ground truth more reproducible in Section 6.2.

### 5.3. Metrics

A few years before Weiler et al. (2019) proposed the artificial stream, they proposed several new metrics. Most adapted the ones that early event tracking literature had adopted from IR research: precision and recall. Like with the artificial stream, Weiler et al. (2015a,b) intended for the new metrics to automate the research area's evaluations and improve reproducibility. Nevertheless, while the new metrics tacitly admit that IR's standard metrics no longer suffice, they continue to depend excessively on precision and recall.

On Twitter, the new language of social media rendered IR's metrics reductive. Evidently, precision and recall did not stop sufficing entirely; by some interpretation, an event too can be precise or imprecise, recalled or missed. Most of Weiler et al. (2015a,b)'s new metrics automated the interpretation and measure of the two. In practice, however, events on Twitter lie on a spectrum of relevance. Precision and recall address the extremes–complete irrelevance and relevance–but not all the other events that lie somewhere in-between.

On this spectrum of relevance, event tracking literature only agrees about what it finds absolutely irrelevant. Spam and advertisements, so rife on Twitter, lie on the outset of the range. Many other events, like opinions and duplicate events, lie somewhere along the spectrum of relevance–not absolutely irrelevant because they relate to the event but neither absolutely relevant like the easily-enumerable events. Still, annotators must decide–precise or imprecise, recalled or missed–and when one study's understanding of precision and recall does not align with the other's, the results become incomparable. We focus, in particular, on the claims to relevance of two classes of events: opinions and redundant events.

Opinions, in particular, divide the research area. An opinion does not fit in the conventional definition of events, “a significant thing that happens at some specific time and place” (McMinn et al., 2013). Even without being events, however, opinions often behave like ones; in document-pivot approaches, they form clusters of tweets with the same sentiment, and in feature-pivot approaches, opinions burst in response to an actual event. Moreover, as McMinn et al. (2013) remarked, opinions are what give allure to Twitter's conversations.

Perhaps the views differ because we cannot agree on what form of media event tracking should emulate: the formal news media or the informal social media. Today, however, even the formal news media's position on opinions seems to be shifting. Punditry has matured into a new and popular form of journalism, The Guardian use opinions to give context to and explain developments (Suárez, 2022), and overall, the news industry seems increasingly-accepting of the idea that journalism could share the same space as opinions (Newman, 2022). Opinions clearly hold some value, unlike spam and advertisements, but the disagreement over whether they constitute precision muddles IR's once-clear metrics.

While the research area appears divided over opinions, it avoids altogether the matter of how many redundant or duplicate events an algorithm captures. In their survey, Weiler et al. (2017) lamented the lack of reporting and discussion on redundancy. Even when Meladianos et al. (2015) designed an algorithm to minimize the number of repeated events, the evaluation methodology did not address redundancy. It seems impossible to reconcile the high precision and recall values of event tracking algorithms with the expectation of capturing no duplicate events. On the contrary, Weiler et al. (2017) found that reporting conceals high rates of duplication.

Redundancy has always accompanied event tracking. The TDT pilot study conceived a research area with a triple role: to segment data streams, to detect events within and, crucially, to track events (Allan et al., 1998a). The lack of reporting about redundancy represents the research community's abandonment of the tracking role. Weiler et al. (2017)'s observed rates of redundancy represent its failure. An event tracking algorithm that detects but does not track cannot aspire to truly minimize information overload. Therefore, the event tracking community should reject redundant events with the same assuredness with which it rejects spam and advertisements. We suggest ways to make event tracking's metrics more reproducible in Section 6.3.

### 5.4. Algorithms

Beyond matters of data, ground truth and metrics lies one more issue whose existence precludes a state-of-the-art: the algorithms. There can be no state-of-the-art without a comparison of algorithms, and comparisons appear scarcely (Weiler et al., 2017). Even when Meladianos et al. (2018) proposed a novel event tracking algorithm to succeed a previous one (Meladianos et al., 2015), they did not compare the new technique with its precursor. When the data changes, and the ground truth and the metrics change, we cannot compare the performance of algorithms across papers. Only direct comparisons, with the same evaluation methodology, will suffice.

Of course, few papers make no comparisons at all. Just 22 studies (27.85%) used no baseline, and 11 others (13.92%) only experimented by tweaking the parameters of the novel algorithms. Nevertheless, of the 36 studies (45.57%) that compared with peer-reviewed algorithms directly, few make meaningful comparisons. Only 15 of those 36 studies (41.67%) compared novel algorithms with at least one baseline published within the previous 2 years. In 13 other studies (36.11%), the most recent baseline had been published four or more years earlier. Of the scarce comparisons, many still give little context to progress.

The relative scarcity of baselines has a simple explanation. Event tracking literature rarely compares algorithms because researchers rarely share algorithms (Guille et al., 2013; Weiler et al., 2015a, 2017). Out of 79 papers, only the five studies in Table 9 open-sourced their algorithms, and the choice tapers quickly. Many techniques show their age in their dated programming languages, or have poor documentation or no easy-to-use interface. Others require obsolete software or a particular type of data, such as tweets collected using a certain version of the Twitter API or formatted in a certain way (Hettiarachchi et al., 2021). If any options remain, they might have been developed for a particular event domain, with designs unsuitable for a study's target domain.

**Table 9:** TDT literature has very few open-source algorithms.

**Publication**	**Language**	**Interface**	**Domains**	**GitHub repository**
Guille et al. (2013)	Java 8	GUI	Unspecified	AdrienGuille/SONDY
Ifrim et al. (2014)	Python 2	CLI	Finance, politics, war	heerme/twitter-topics
Van Canneyt et al. (2014)	Java	CLI	Finance, politics, war	svcanney/twittertopics
Hettiarachchi et al. (2021)	Python 3	CLI	Football, politics	HHansi/Embed2Detect
Mamo et al. (2021)	Python 3	CLI	Football	NicholasMamo/eld-data

Practical considerations, such as the need for an algorithm designed for a particular domain, whittles down the choice of baseline even further.

In the face of such difficulties, many implemented their own baselines. They implemented basic algorithms, the “naïve” (Buntain et al., 2016) and simplistic (Hsu et al., 2018) volume-based methods of early event tracking research: Marcus et al. (2011)'s, Zhao et al. (2011)'s, and Vasudevan et al. (2013)'s. Six studies implemented (Petrović et al., 2010, 2012)'s LSH algorithm (Aiello et al., 2013; McMinn and Jose, 2015; Preoţiuc-Pietro et al., 2016; Li et al., 2017; Hasan et al., 2019; Kolajo et al., 2022) and tellingly, all six implemented the first version from 2010 (Petrović et al., 2010). The 2012 version (Petrović et al., 2012) grew in sophistication and performance, but it grew in complexity too.

Implementing baselines will not solve the research area's issues of reproducibility. Manual efforts reassert themselves, this time to deter the prospects of implementing someone else's solution (McMinn et al., 2013). No one knows the nuanced design of an algorithm better than its author, and innocuous modifications can drastically affect results (Weiler et al., 2016; Raff, 2019). Thus, the simplest of techniques become pretend-baselines to establish a false state-of-the-art. In the next section, we suggest how event tracking evaluations can be made more reproducible.

## 6. Discussion

Twitter (2020a)'s data sharing policy weighs heavy on the issues of reproducibility, but what most impressed us in conducting this review is just how few of the issues relate to it. Most issues originate unforced from the researchers' own decisions. The flaws of manual and automatic evaluation methodologies cannot be allowed to excuse irreproducible practices. In this section, we suggest how event tracking literature can make its evaluations more reproducible despite Twitter's policy.

### 6.1. Data

Our brief experiment in Section 5.1 leaves little doubt: dataset reuse in its current form cannot and will not lead to establishing a state-of-the-art. Twitter's policy relegated the ideal evaluation to a past in which social networks did not exist. Faced with these new realities, the practice of dataset reuse must cease or risk misleading event tracking literature further. Nevertheless, Twitter's policy accounts for few of the research area's evaluation challenges. We make three suggestions for event tracking literature to improve data reproducibility.

Our first suggestion is to vary datasets, and to describe them both quantitatively and qualitatively. Researchers often reveal little information about their corpora. We observed researchers gloss over how they collected datasets (Meladianos et al., 2018), how many tweets they collected (Aiello et al., 2013) or how many remained available to download (Choi and Park, 2019), and any notable characteristics that may prejudice results, like the levels of noise. Moreover, researchers generally used few datasets, with data too scarce and too unvaried to draw generalizable conclusions. In this regard, at least, Meladianos et al. (2018)'s evaluation stands as a model: 18 varied events, popular and unpopular, enabled by a partially-automated evaluation methodology.

Our second suggestion is to explore whether artificial data could replace the real data missing from tweet datasets. Now we know what the missing tweets characterize: noise. Had we missed events or opinions, we would have had to surmise Who did What, Where and When, or what opinion a user holds to regenerate the lost tweets, but noise is noise. The subject matter of noise changes little and matters little. In fact, we understand noise and spam so well that Hasan et al. (2019) could manually curate a list of 350 spam phrases. Because we understand noise so well, we might be able to re-inject artificial noise back into datasets without having to replicate the original, lost noise.

Our third suggestion is to experiment with different types of datasets. The missing, noisy data could transform into an asset to evaluations. Repeated experiments could reveal how well an algorithm handles noise by comparing its performance on the original and re-downloaded datasets. Weiler et al. (2019)'s artificial stream could also play a role. It would be presumptuous to discard tweets entirely from the evaluation process, but the artificial stream could coexist with traditional tweet corpora. Event tracking literature could combine datasets: automatic evaluations on a shared set of Weiler et al. (2019)'s artificial datasets and manual evaluations on real tweet corpora. On the former, event tracking literature could establish a tentative state-of-the-art. On the latter, it could provide real-world context to results.

### 6.2. Ground truth

A reproducible ground truth will not establish a state-of-the-art, but it can reveal the general state of event tracking. In the domain of football matches, consistent ground truths have allowed us to uncover a steady pattern, how algorithms capture major events more comfortably than minor events (Marcus et al., 2011; Nichols et al., 2012; Meladianos et al., 2015, 2018). To draw such patterns, however, researchers must reject the liberty that they have allowed to infect event tracking's evaluations. We make two suggestions for event tracking literature to improve ground truth reproducibility.

Our first suggestion is to develop a new theory of events. Reproducible ground truths start with systematic annotations, and systematic annotations start with a common definition of events. If the research community cannot agree on what defines an event, then we cannot expect it to agree on what defines a ground truth event. Event tracking literature might find a definition nearby, in event modeling research. In event modeling, researchers could not represent events formally without a structured definition of events, which some found in the ‘four Ws', or *Who* did *What, Where* and *When* (Chen and Li, 2020). To expect event tracking algorithms to model events might appear unrealistic, but we could express the ground truth in terms of the “four Ws.” Similarly to Zhou et al. (2015, 2017), we could expect a document-pivot technique's clusters to describe Who did What, Where and When, or a feature-pivot technique's keywords to capture them.

The latter describes, crudely, keyword matching evaluations, whose ground truths too lack reproducibility. Consider how Aiello et al. (2013) and Hettiarachchi et al. (2021) evaluated. Consider how the manual selection of ground truth keywords imparts subjectivity on a process meant to typify objectivity. We could only find one attempt to construct a keyword-based ground truth automatically. Zhang et al. (2021) elected nouns from authoritative news accounts on Twitter using Term Frequency-Inverse Document Frequency (TF-IDF). Regrettably, however, Zhang et al. (2021) failed to show that TF-IDF produces a reliable ground truth from the brevity that characterizes tweets and headlines (Marujo et al., 2015).

Our second suggestion is to construct keyword ground truths automatically, but more robustly and less subjectively with keyphrase extraction algorithms. Algorithms like Campos et al. (2020)'s YAKE! could automatically extract the ground truth keywords from minute-by-minute reports, headlines or tweets. YAKE! outperformed other keyphrase extraction algorithms on short texts (Campos et al., 2020), and our brief experiments in Table 10 show how YAKE!'s output correlates closely with Aiello et al. (2013)'s manual judgments. More than just eliminating human error and bias, keyphrase extraction scales better. Freed from the burden of manually-curating lists of keywords, evaluations can combine ground truths from multiple sources to capture more lexical and stylistic variety.

**Table 10:** Keyphrase extraction can replace manual annotation in keyword evaluations.

**Topic**	**Keywords (Aiello et al., 2013)**	**YAKE! (Campos et al., 2020)**
Chelsea 1-0 Liverpool Ramires scores a goal from inside the box to the bottom left corner of the goal.	Ramires, goal, 1-0, Chelsea, score, yes	Chelsea, Liverpool, Ramires, goal, scores
Newt Gingrich: “Thank you Georgia! It is gratifying to win my home state so decisively to launch our March Momentum”	Newt, Gingrich, thank, Georgia, March, Momentum, gratifying	Gingrich, Georgia, Newt, Momentum, March
Republican Party keeps control of the House of Representatives	GOP, Republican, House, control	Representatives, Party, House, Republican, control

Above, Campos et al. (2020)'s YAKE! is set to extract the five highest-scoring unigram keyphrases from the topic's description.

### 6.3. Metrics

Weiler et al. (2015a,b)'s metrics did not only automate precision and recall. Two other metrics, *throughput* and *redundancy*, measured aspects radically different from what precision and recall captured: how efficient an algorithm and how well it tracked events. Our suggestion for more reproducible metrics follows in the same path as Weiler et al. (2015a,b)'s. We argue that event tracking needs a more systematic, nuanced interpretation of precision.

Precision forced researchers into a false dichotomy: precise or imprecise. Opinions and redundant events do not have to be precise or imprecise. Opinions and redundant events can simply be opinions and redundant events. In other words, like Weiler et al. (2015a,b)'s redundancy metric, research should ask whether an event is an opinion or not, and whether it is redundant or not, rather than coerce the two types of topics into a simplified representation of precision. Specifically, we propose four types of labels that capture the new aspects of social media content: precise ground truth events, opinions, redundant events and noise.

### 6.4. Algorithms

The matter of reproducible algorithms has a simpler solution. Given everything that we know about datasets and dataset sharing, and the subjectivity seemingly-ingrained in our ground truths and metrics, our suggestion to improve the reproducibility of algorithms is a simple one: publicly-available algorithms. Algorithms need to be readily-available, accept standard inputs–raw tweets–and produce standard outputs, document their implementation clearly and offer intuitive interfaces to tweak parameters. Few such algorithms emerged during our review.

Evidently, open-source solutions by themselves will not extinguish human error and bias. In this way too, the SNOW 2014 Data Challenge remains a gold standard (Papadopoulos et al., 2014). The annotators did not have access to the algorithms–only to their standardized outputs–but they applied their bias consistently. Likewise, open-source algorithms would allow the event tracking community to apply subjectivity uniformly to baselines and novel algorithms. Only then may a more concrete semblance of a state-of-the-art materialize. To this end, we plan to release an extensible library to facilitate the development and sharing of event tracking algorithms.

## 7. Conclusion

There can be no state-of-the-art without a reproducible evaluation methodology. In this review, we demonstrated the absence of reproducibility in event tracking's evaluations. Twitter's data sharing policy relegated the ideal IR evaluation to history, even as event tracking toiled helplessly. What remains of the research area's efforts are scattered indicators of progress. Manual evaluations failed, and so did automatic alternatives. Event tracking may be doomed never to replicate the ideally-reproducible evaluations of early TDT research (Allan et al., 1998a), at least on Twitter.

Event tracking still needs a reproducible evaluation methodology, today perhaps more than ever, and not only for the sake of a state-of-the-art. The research area of event modeling and mining (Chen and Li, 2020), a successor to formalize event tracking's outputs, suffers from the same issues of reproducibility; its research community too evaluates models indirectly, often through the event tracking algorithm's own outputs. Elsewhere, event tracking has started to aid newsrooms in newsgathering efforts (Beckett, 2019; Newman, 2022), but the modern newsroom cannot harness event tracking without a measure of progress.

Our review echoes (Weiler et al., 2017)'s sentiment. Our suggestions can improve reproducibility, but only if literature reserves an individual answer to the complex matter of how to evaluate. Event tracking research needs new forms of data to remedy corrupted tweet corpora, a new theory of events with which to build the ground truth and new metrics with which to apply it, and a new tradition of openness in sharing algorithms. In other words, event tracking literature must approach its evaluations as an independent research question. Researchers must acknowledge that Twitter's policy hinders their evaluations, but so do their decisions.

## Author contributions

NM, JA, and CL: conceptualization, methodology, resources, writing—original draft preparation, and writing—review and editing. NM: software, validation, formal analysis, investigation, data curation, and funding acquisition. JA and CL: supervision and project administration. All authors have read and agreed to the published version of the manuscript.
