# Supplementary material for: The myth of reproducibility: A review of event tracking evaluations on Twitter
Source: Front Big Data. 2023 Apr 5;6:1067335. doi: 10.3389/fdata.2023.1067335 (PMC10113524; doi:10.3389/fdata.2023.1067335)
Supplement: Supplementary file 1 [file Table_1.PDF]

frontiers

Supplementary Material

This supplementary material lists the 79 publications that we reviewed. Alongside each publication, Table S1 includes a summary of the most important properties: the type of evaluation, who annotated the datasets, the number and sizes of datasets, and the types of baselines that they used. Note that we only filled in cells in the table when researchers made their evaluation methods explicit. Furthermore, the annotator and baseline columns may contain multiple types of values. The main text includes tables with more fine-grained details about smaller selections of publications.

Table S1: A summary of event tracking evaluation methodologies on Twitter, including analyses of the event tracking components in event modelling and mining architectures. We only filled in the data for publications that made their approaches explicit.

Publication	Evaluation	Annotators	Datasets	Tweets	Baseline
Sankaranarayanan et al. (2009)	None				None
Petrović et al. (2010)	Manual	External			Published algorithms, parameter tweaking, trivial baselines
Phuvipadawat and Murata (2010)	Empirical				Parameter tweaking
Sakaki et al. (2010)	Manual		2		None
Chakrabarti and Punera (2011)	Manual	External			Parameter tweaking
Choudhury and Breslin (2011)	Semi-automatic	External	1	1,500	None
Earle et al. (2011)	Manual	Researchers			None
Gu et al. (2011)	Manual	Researchers and external			Published algorithms, parameter tweaking
Lanagan and Smeaton (2011)	Manual	External	8	101–4,073	Parameter tweaking
Marcus et al. (2011)	Manual	Researchers	4		None
Popescu et al. (2011)	Semi-automatic				Parameter tweaking
Shamma et al. (2011)	Empirical		2		None
Zhao et al. (2011)			33		Parameter tweaking
Hsieh et al. (2012)	Manual	Researchers	18	81,655–1,455,151	None
Nichols et al. (2012)	Manual	Researchers	3	72,335–113,189	None
Ozdikis et al. (2012)	Manual	Researchers			Parameter tweaking
Petrović et al. (2012)	Semi-automatic	External		50,000,000	Published algorithms, parameter tweaking, published results
van Oorschot et al. (2012)	Automatic		63	1,050,343	Parameter tweaking
Aiello et al. (2013)	Semi-automatic	Researchers	3		Published algorithms
Cataldi et al. (2013)	Manual	External			None
Guille et al. (2013)	Empirical	External		7,874,772	None
Shen et al. (2013)	Semi-automatic	External	7	163,775–345,335	Published algorithms
Vasudevan et al. (2013)			15	3,000,000	None
Burnside et al. (2014)	Manual	External	4		Published results
Chierichetti et al. (2014)	Automatic		2	1,490,000–342,000,000	Trivial baselines
Corney et al. (2014)	Semi-automatic	Researchers	2		None

Publication	Evaluation	Annotators	Datasets	Tweets	Baseline
Guille and Favre (2014)	Manual	External	2	1,437,126–2,086,136	Published algorithms, parameter tweaking
Ifrim et al. (2014)	Manual	External	4		Published results
Martin-Dancausa and Göker (2014)	Manual	External	4		Published results
Nutakki et al. (2014)	Manual	External	4		Published results
Petkos et al. (2014)	Empirical				None
Van Canneyt et al. (2014)	Manual	External	4		Published results
De Boom et al. (2015)	Semi-automatic	Researchers			Parameter tweaking
Feng et al. (2015)	Manual	External		9,000,000	Published algorithms
Kumar et al. (2015)	Manual	Researchers	2		None
Löchtefeld et al. (2015)	Manual		42	103–11,894	None
Madani et al. (2015)	Manual	Researchers			Published algorithms
McMinn and Jose (2015)	Manual	External			Published algorithms
Meladianos et al. (2015)	Semi-automatic	External	13	72,335–1,907,999	Trivial baselines
Unankard et al. (2015)	Manual	Researchers	1	196,834	Published algorithms
Weiler et al. (2015a)					Published algorithms, trivial baselines
Weiler et al. (2015b)					Published algorithms, trivial baselines
Zhou et al. (2015)	Manual		2		Published algorithms
Adedoyin-Olowe et al. (2016)	Manual	Researchers	3		None
Buntain et al. (2016)			3	809,426–1,166,767	Published algorithms
Hua et al. (2016)					Published algorithms
Liu et al. (2016)	Semi-automatic			357,000,000	Published algorithms, parameter tweaking
Preoţiuc-Pietro et al. (2016)	Semi-automatic	External	3	2,500,000–150,000,000	Published algorithms, parameter tweaking
Weiler et al. (2016)	Automatic				Published algorithms
Zhou et al. (2016)	None				None
Akhtar and Siddique (2017)			1	58,000	Parameter tweaking
Edouard et al. (2017)	Automatic			2,342–43,000,000	Published algorithms
Hammad and El-Beltagy (2017)	Manual	Researchers			None
Li et al. (2017)	Semi-automatic	External			Published algorithms
Liu et al. (2017)	Manual	External			Published algorithms

Publication	Evaluation	Annotators	Datasets	Tweets	Baseline
Mamo and Azzopardi (2017)	Manual	Researchers			Published algorithms
Nolasco and Oliveira (2017)	Manual		1	432,975	None
Tonon et al. (2017)	Manual	Researchers		195,700,000	None
Weiler et al. (2017)	Manual	Researchers			Published algorithms, trivial baselines
Zhou et al. (2017)	Manual		2		Published algorithms
Chen and Terejanu (2018)			3		Parameter tweaking
Hossny and Mitchell (2018)					Published algorithms
Huang et al. (2018)	Semi-automatic	External	5	218,313–345,335	Published algorithms
Meladianos et al. (2018)	Semi-automatic	External	20	41,539–973,985	Trivial baselines
Petroni et al. (2018)	None			25,000	None
Choi and Park (2019)	Automatic		3		Published results
GabAllah and Rafea (2019)	Manual				Published algorithms
Hasan et al. (2019)	Manual	External			Published algorithms
Mele et al. (2019)	Manual	External		80,134	Published algorithms
Pradhan et al. (2019)	Manual	External	3	1,653–24,667	None
Saeed et al. (2019)	Automatic		3	124,524–2,335,105	Published results
Weiler et al. (2019)	Automatic				Published algorithms
Farnaghi et al. (2020)	Automatic		1		Parameter tweaking
George et al. (2021)	Manual	Researchers		203,519	Published algorithms, trivial baselines
Hettiarachchi et al. (2021)	Semi-automatic	Researchers		99,995–174,498	Published algorithms, parameter tweaking
Mamo et al. (2021)	Manual	Researchers	6	63,891–303,982	Published algorithms
Zhang et al. (2021)	Automatic				Published algorithms, trivial baselines
Di Corso et al. (2022)	Automatic		6	60,005	Published algorithms, parameter tweaking
Kolajo et al. (2022)	Automatic			82,887	Published algorithms

REFERENCES

Adedoyin-Olowe, M., Gaber, M. M., Dancausa, C. M., Stahl, F., and Gomes, J. B. (2016). A Rule Dynamics Approach to Event Detection in Twitter with its Application to Sports and Politics. *Expert Systems with Applications* 55, 351–360

Aiello, L. M., Petkos, G., Martin, C., Corney, D., Papadopoulos, S., Skraba, R., et al. (2013). Sensing Trending Topics in Twitter. *IEEE Transactions on Multimedia* 15, 1268–1282

Akhtar, N. and Siddique, B. (2017). Hierarchical Visualization of Sport Events Using Twitter. *Journal of Intelligent & Fuzzy Systems* 32, 2953–2961

Buntain, C., Lin, J., and Golbeck, J. (2016). Discovering Key Moments in Social Media Streams. In *2016 13th IEEE Annual Consumer Communications & Networking Conference (CCNC)* (Las Vegas, NV, USA: IEEE), 366–374

Burnside, G., Milioris, D., and Jacquet, P. (2014). One Day in Twitter: Topic Detection Via Joint Complexity. In *Proceedings of the SNOW 2014 Data Challenge* (Seoul, Korea: CEUR), 41–48

Cataldi, M., Caro, L. D., and Schifanella, C. (2013). Personalized Emerging Topic Detection Based on a Term Aging Model. *ACM Transactions on Intelligent Systems and Technology (TIST)* 5, 1–27

Chakrabarti, D. and Punera, K. (2011). Event Summarization Using Tweets. In *Proceedings of the Fifth International AAAI Conference on Weblogs and Social Media* (Barcelona, Spain: The AAAI Press), 66–73

Chen, C. and Terejanu, G. (2018). Sub-Event Detection on Twitter Network. In *AIAI 2018: AIAI: 14th IFIP International Conference on Artificial Intelligence Applications and Innovations* (Rhodes, Greece: Springer International Publishing), 50–60

Chierichetti, F., Kleinberg, J., Kumar, R., Mahdian, M., and Pandey, S. (2014). Event Detection via Communication Pattern Analysis. In *Proceedings of the Eighth International AAAI Conference on Weblogs and Social Media* (Ann Arbor, MI, USA: Association for the Advancement of Artificial Intelligence), 51–60

Choi, H.-J. and Park, C. H. (2019). Emerging Topic Detection in Twitter Stream Based on High Utility Pattern Mining. *Expert Systems with Applications* 115, 27–36

Choudhury, S. and Breslin, J. G. (2011). Extracting Semantic Entities and Events from Sports Tweets. In *Proceedings of the ESWC2011 Workshop on 'Making Sense of Microposts': Big Things Come in Small Packages* (Heraklion, Crete), 22–32

Corney, D., Martin, C., and Göker, A. (2014). Spot the Ball: Detecting Sports Events on Twitter. In *ECIR 2014: Advances in Information Retrieval* (Amsterdam, The Netherlands: Springer), 449–454

De Boom, C., Van Canneyt, S., and Dhoedt, B. (2015). Semantics-Driven Event Clustering in Twitter feeds. In *Proceedings of the 5th Workshop on Making Sense of Microposts* (Florence, Italy: CEUR), 2–9

Di Corso, E., Proto, S., Vacchetti, B., Bethaz, P., and Cerquitelli, T. (2022). Simplifying Text Mining Activities: Scalable and Self-Tuning Methodology for Topic Detection and Characterization. *Applied Sciences* 12, 1–41

Earle, P. S., Bowden, D., and Guy, M. (2011). Twitter Earthquake Detection: Earthquake Monitoring in a Social World. *Annals of Geophysics* 54, 708–715

Edouard, A., Cabrio, E., Tonelli, S., and Le Thanh, N. (2017). Graph-Based Event Extraction from Twitter. In *Proceedings of the International Conference Recent Advances in Natural Language Processing, RANLP 2017* (Varna, Bulgaria: INCOMA Ltd.), 222–230

Farnaghi, M., Ghaemi, Z., and Mansourian, A. (2020). Dynamic Spatio-Temporal Tweet Mining for Event Detection: A Case Study of Hurricane Florence. *International Journal of Disaster Risk Science* 11, 378–393

Feng, W., Zhang, C., Zhang, W., Han, J., Wang, J., Aggarwal, C., et al. (2015). STREAMCUBE: Hierarchical Spatio-Temporal Hashtag Clustering for Event Exploration over the Twitter Stream. In *2015 IEEE 31st International Conference on Data Engineering* (Seoul, South Korea: IEEE), 1561–1572

GabAllah, N. and Rafea, A. (2019). Unsupervised Topic Extraction from Twitter: A Feature-pivot Approach. In *Proceedings of the 15th International Conference on Web Information Systems and Technologies - Volume 1: WEBIST* (Vienna, Austria: SCITEPRESS – Science and Technology Publications), 185–192

George, Y., Karunasekera, S., Harwood, A., and Lim, K. H. (2021). Real-Time Spatio-Temporal Event Detection on Geotagged Social Media. *Journal of Big Data* 8, 1–28

Gu, H., Xie, X., Lv, Q., Ruan, Y., and Shang, L. (2011). ETree: Effective and Efficient Event Modeling for Real-Time Online Social Media Networks . In *WI-IAT '11: Proceedings of the 2011 IEEE/WIC/ACM International Conferences on Web Intelligence and Intelligent Agent Technology - Volume 01* (Lyon, France: IEEE Computer Society), 300–307

Guille, A. and Favre, C. (2014). Mention-Anomaly-Based Event Detection and Tracking in Twitter. In *Proceedings of the 2014 IEEE/ACM International Conference on Advances in Social Network Analysis and Mining* (Beijing, China: IEEE), 375–382

Guille, A., Favre, C., Hacid, H., and Zighed, D. (2013). SONDY: An Open Source Platform for Social Dynamics Mining and Analysis. In *Proceedings of the 2013 ACM SIGMOD International Conference on Management of Data* (New York, NY, USA: Association for Computing Machinery), 1005–1008

Hammad, M. and El-Beltagy, S. R. (2017). Towards Efficient Online Topic Detection through Automated Bursty Feature Detection from Arabic Twitter Streams. *Procedia Computer Science* 117, 248–255

Hasan, M., Orgun, M. A., and Schwitter, R. (2019). Real-Time Event Detection from the Twitter Data Stream Using the TwitterNews+ Framework. *Information Processing & Management* 56, 1146–1165

Hettiarachchi, H., Adedoyin-Olowe, M., Bhogal, J., and Gaber, M. M. (2021). Embed2Detect: Temporally Clustered Embedded Words for Event Detection in Social Media. *Machine Learning* 111, 49–87

Hossny, A. H. and Mitchell, L. (2018). Event Detection in Twitter: A Keyword Volume Approach. In *2018 IEEE International Conference on Data Mining Workshops (ICDMW)* (Singapore: IEEE), 1200–1208

Hsieh, L.-C., Lee, C.-W., Chiu, T.-H., and Hsu, W. (2012). Live Semantic Sport Highlight Detection Based on Analyzing Tweets of Twitter. In *2012 IEEE International Conference on Multimedia and Expo* (Melbourne, VIC, Australia: IEEE), 949–954

Hua, T., Chen, F., Zhao, L., Lu, C.-T., and Ramakrishnan, N. (2016). Automatic Targeted-Domain Spatiotemporal Event Detection in Twitter. *GeoInformatica* 20, 765–795

Huang, Y., Shen, C., and Li, T. (2018). Event Summarization for Sports Games using Twitter Streams. *World Wide Web* 21, 609–627

Ifrim, G., Shi, B., and Brigadir, I. (2014). Event Detection in Twitter using Aggressive Filtering and Hierarchical Tweet Clustering. In *Proceedings of the SNOW 2014 Data Challenge* (Seoul, Korea: CEUR), 33–40

Kolajo, T., Daramola, O., and Adebiyi, A. A. (2022). Real-Time Event Detection in Social Media Streams Through Semantic Analysis of Noisy Terms. *Journal of Big Data* 9, 1–36

Kumar, S., Liu, H., Mehta, S., and Subramaniam, L. V. (2015). Exploring a Scalable Solution to Identifying Events in Noisy Twitter Streams. In *Proceedings of the 2015 IEEE/ACM International Conference on Advances in Social Networks Analysis and Mining 2015* (Paris, France: IEEE), 496–499

Lanagan, J. and Smeaton, A. F. (2011). Using Twitter to Detect and Tag Important Events in Live Sports. In *Proceedings of the Fifth International AAAI Conference on Weblogs and Social Media* (Barcelona, Spain: Association for the Advancement of Artificial Intelligence), 542–545

Li, Q., Nourbakhsh, A., Shah, S., and Liu, X. (2017). Real-Time Novel Event Detection from Social Media. In *2017 IEEE 33rd International Conference on Data Engineering (ICDE)* (San Diego, CA, USA: IEEE), 1129–1139

Liu, X., Li, Q., Nourbakhsh, A., Fang, R., Thomas, M., Anderson, K., et al. (2016). Reuters Tracer: A Large Scale System of Detecting & Verifying Real-Time News Events from Twitter. In *CIKM '16: Proceedings of the 25th ACM International Conference on Information and Knowledge Management* (Indianapolis, IN, USA: Association for Computing Machinery), 207–216

Liu, X., Nourbakhsh, A., Li, Q., Shah, S., Martin, R., and Duprey, J. (2017). Reuters Tracer: Toward Automated News Production Using Large Scale Social Media Data. In *2017 IEEE International Conference on Big Data (Big Data)* (Boston, MA, USA: IEEE), 1483–1493

Löchtefeld, M., Jäckel, C., and Krüger, A. (2015). TwitSoccer: Knowledge-Based Crowd-Sourcing of Live Soccer Events. In *MUM '15: Proceedings of the 14th International Conference on Mobile and Ubiquitous Multimedia* (Linz, Austria: ACM), 148–151

Madani, A., Boussaid, O., and Zegour, D. (2015). Real-Time Trending Topics Detection and Description from Twitter Content. *Social Network Analysis and Mining* 5, 1–13

Mamo, N. and Azzopardi, J. (2017). FIRE: Finding Important News REports. In *Semantic Keyword-Based Search on Structured Data Sources*, eds. J. Szymański and Y. Velegrakis (Gdansk, Poland: Springer International Publishing), 20–31

Mamo, N., Azzopardi, J., and Layfield, C. (2021). Fine-grained Topic Detection and Tracking on Twitter. In *Proceedings of the 13th International Joint Conference on Knowledge Discovery, Knowledge Engineering and Knowledge Management - (Volume 1)* (Remote: SciTePress), 79–86

Marcus, A., Bernstein, M., Badar, O., Karger, D., Madden, S., and Miller, R. (2011). Twitinfo: Aggregating and Visualizing Microblogs for Event Exploration. In *CHI '11: Proceedings of the SIGCHI Conference on Human Factors in Computing Systems* (Vancouver, BC, Canada.: Association for Computing Machinery), 227–236. May 7–12, 2011

Martin-Dancausa, C. and Göker, A. (2014). Real-time Topic Detection with Bursty N-Grams. In *Proceedings of the SNOW 2014 Data Challenge co-located with 23rd International World Wide Web Conference (WWW 2014)* (Seoul, Korea: CEUR), 9–16

McMinn, A. J. and Jose, J. M. (2015). Real-Time Entity-Based Event Detection for Twitter. In *CLEF 2015: Experimental IR Meets Multilinguality, Multimodality, and Interaction*, eds. J. Mothe, J. Savoy, J. Kamps, Pinel-Sa, K. Pinel-Sauvagnat, G. Jones, E. San Juan, L. Capellato, and F. Nicola (Toulouse, France: Springer International Publishing), 65–77

Meladianos, P., Nikolentzos, G., Rousseau, F., Stavrakas, Y., and Vazirgiannis, M. (2015). Degeneracy-Based Real-Time Sub-Event Detection in Twitter Stream. In *Proceedings of the Ninth International AAAI Conference on Web and Social Media* (Oxford, United Kingdom: The AAAI Press), 248–257

Meladianos, P., Xypolopoulos, C., Nikolentzos, G., and Vazirgiannis, M. (2018). An Optimization Approach for Sub-Event Detection and Summarization in Twitter. In *Advances in Information Retrieval* (Grenoble, France: Springer International Publishing), 481–493

Mele, I., Bahrainian, S. A., and Crestani, F. (2019). Event Mining and Timeliness Analysis from Heterogeneous News Streams. *Information Processing & Management* 56, 969–993

Nichols, J., Mahmud, J., and Drews, C. (2012). Summarizing Sporting Events Using Twitter. In *Proceedings of the 2012 ACM International Conference on Intelligent User Interfaces* (Lisbon, Portugal: ACM), 189–198

Nolasco, D. and Oliveira, J. (2017). Intelligent Subevent Detection Based on Social Network Data. In *2017 IEEE 15th Intl Conf on Dependable, Autonomic and Secure Computing, 15th Intl Conf on Pervasive Intelligence and Computing, 3rd Intl Conf on Big Data Intelligence and Computing and Cyber Science and Technology Congress(DASC/PiCom/DataCom/CyberSciTech)* (Orlando, FL, USA: IEEE), 820–827

Nutakki, G. C., Nasraoui, O., Abdollahi, B., Badami, M., and Sun, W. (2014). Distributed LDA Based Topic Modeling and Topic Agglomeration in a Latent Space. In *Proceedings of the SNOW 2014 Data Challenge* (Seoul, Korea: CEUR), 17–24

Ozdikis, O., Senkul, P., and Oguztuzun, H. (2012). Semantic Expansion of Tweet Contents for Enhanced Event Detection in Twitter. In *2012 IEEE/ACM International Conference on Advances in Social Networks Analysis and Mining* (Istanbul, Turkey: IEEE), 20–24

Petkos, G., Papadopoulos, S., and Kompatsiaris, Y. (2014). Two-Level Message Clustering for Topic Detection in Twitter. In *Proceedings of the SNOW 2014 Data Challenge* (Seoul, Korea: CEUR), 49–56

Petroni, F., Raman, N., Nugent, T., Nourbakhsh, A., Panić, Z., Shah, S., et al. (2018). An Extensible Event Extraction System With Cross-Media Event Resolution. In *KDD '18: Proceedings of the 24th ACM SIGKDD International Conference on Knowledge Discovery & Data Mining* (London, United Kingdom: Association for Computing Machinery), 626–635

Petrović, S., Osborne, M., and Lavrenko, V. (2010). Streaming First Story Detection with Application to Twitter. In *HLT '10: Human Language Technologies: The 2010 Annual Conference of the North American Chapter of the Association for Computational Linguistics* (Los Angeles, CA, United States of America: Association for Computational Linguistics), 181–189

Petrović, S., Osborne, M., and Lavrenko, V. (2012). Using Paraphrases for Improving First Story Detection in News and Twitter. In *NAACL HLT '12: Proceedings of the 2012 Conference of the North American Chapter of the Association for Computational Linguistics: Human Language Technologies* (Montreal, Canada: Association for Computational Linguistics), 338–.346

Phuvipadawat, S. and Murata, T. (2010). Breaking News Detection and Tracking in Twitter. In *Proceedings of the 2010 IEEE/WIC/ACM International Conference on Web Intelligence and Intelligent Agent Technology* (Toronto, ON, Canada: IEEE Computer Society), 120–123

Popescu, A.-M., Pennacchiotti, M., and Paranjpe, D. (2011). Extracting Events and Event Descriptions from Twitter. In *Proceedings of the 20th International Conference Companion on World Wide Web* (Hyderabad, India: ACM), 105–106

Pradhan, A. K., Mohanty, H., and Lal, R. P. (2019). Event Detection and Aspects in Twitter: A BoW Approach. In *ICDCIT 2019: Proceedings of the 15th International Conference on Distributed Computing and Internet Technology* (Bhubaneswar, India: Springer International Publishing), 194–211

Preoţiuc-Pietro, D., Srijith, P. K., Hepple, M., and Cohn, T. (2016). Studying the Temporal Dynamics of Word Co-Occurrences: An Application to Event Detection. In *Proceedings of the Tenth International Conference on Language Resources and Evaluation (LREC'16)* (Portorož, Slovenia: European Language Resources Association (ELRA)), 4380–4387

Saeed, Z., Abbasi, R. A., Razzak, I., Maqbool, O., Sadaf, A., and Xu, G. (2019). Enhanced Heartbeat Graph for Emerging Event Detection on Twitter using Time Series Networks. *Expert Systems with Applications* 136, 115–132

Sakaki, T., Okazaki, M., and Matsuo, Y. (2010). Earthquake Shakes Twitter Users: Real-time Event Detection by Social Sensors. In *WWW '10: Proceedings of the 19th International Conference on World Wide Web* (Raleigh, North Carolina, USA: Association for Computing Machinery), 851–860

Sankaranarayanan, J., Samet, H., Teitler, B., Lieberman, M., and Sperling, J. (2009). TwitterStand: News in Tweets . In *GIS '09: Proceedings of the 17th ACM SIGSPATIAL International Conference on Advances in Geographic Information Systems* (Seattle, WA, USA: Association for Computing Machinery), 42–51

Shamma, D., Kennedy, L., and Churchill, E. (2011). Peaks and Persistence: Modeling the Shape of Microblog Conversations. In *CSCW'11: Proceedings of the ACM 2011 Conference on Computer Supported Cooperative Work* (Hangzhou, China: Association for Computing Machinery), 355–358

Shen, C., Liu, F., Weng, F., and Li, T. (2013). A Participant-Based Approach for Event Summarization Using Twitter Streams. In *Proceedings of the 2013 Conference of the North American Chapter of the Association for Computational Linguistics: Human Language Technologies* (Atlanta, Georgia, USA: Association for Computational Linguistics), 1152–1162

Tonon, A., Cudré-Mauroux, P., Blarer, A., Lenders, V., and Motik, B. (2017). ArmaTweet: Detecting Events by Semantic Tweet Analysis. In *The Semantic Web: 14th International Conference, ESWC 2017* (Portorož, Slovenia: Springer International Publishing), 138–153

Unankard, S., Li, X., and Sharaf, M. A. (2015). Emerging Event Detection in Social Networks with Location Sensitivity. *World Wide Web* 18, 1393–1417

Van Canneyt, S., Feys, M., Schockaert, S., Demeester, T., Develder, C., and Dhoedt, B. (2014). Detecting Newsworthy Topics in Twitter. In *Proceedings of the SNOW 2014 Data Challenge* (Seoul, Korea: CEUR), 25–32

van Oorschot, G., van Erp, M., and Dijkshoorn, C. (2012). Automatic Extraction of Soccer Game Events from Twitter. In *Proceedings of the Workshop on Detection, Representation, and Exploitation of Events in the Semantic Web (DeRiVE 2012)* (Boston, Massachusetts, USA: CEUR), 21–30

Vasudevan, V., Wickramasuriya, J., Zhao, S., and Zhong, L. (2013). Is Twitter a Good Enough Social Sensor for Sports TV? . In *2013 IEEE International Conference on Pervasive Computing and Communications Workshops (PERCOM Workshops)* (San Diego, CA, USA: IEEE), 181–186

Weiler, A., Beel, J., Gipp, B., and Grossniklaus, M. (2016). Stability Evaluation of Event Detection Techniques for Twitter. In *Lecture Notes in Computer Science book series (LNCS, volume 9897)* (Stockholm, Sweden: Springer), 368–380

Weiler, A., Grossniklaus, M., and Scholl, M. H. (2015a). Evaluation Measures for Event Detection Techniques on Twitter Data Streams. In *Lecture Notes in Computer Science book series (LNCS, volume 9147)* (Edinburgh, UK: Springer), 108–119

Weiler, A., Grossniklaus, M., and Scholl, M. H. (2015b). Run-Time and Task-Based Performance of Event Detection Techniques for Twitter. In *Lecture Notes in Computer Science book series (LNCS, volume 9097)* (Stockholm, Sweden: Springer International Publishing), 35–49

Weiler, A., Grossniklaus, M., and Scholl, M. H. (2017). Survey and Experimental Analysis of Event Detection Techniques for Twitter. *The Computer Journal* 60, 329–346

Weiler, A., Schilling, H., Kircher, L., and Grossniklaus, M. (2019). Towards Reproducible Research of Event Detection Techniques for Twitter. In *2019 6th Swiss Conference on Data Science (SDS)* (Bern, Switzerland: IEEE), 69–74

Zhang, Y., Shirakawa, M., and Hara, T. (2021). A General Method for Event Detection on Social Media. In *Advances in Databases and Information Systems: 25th European Conference, ADBIS 2021* (Tartu, Estonia: Springer Cham), 43–56

Zhao, S., Zhong, L., Wickramasuriya, J., and Vasudevan, V. (2011). *Human as Real-Time Sensors of Social and Physical Events: A Case Study of Twitter and Sports Games*. Tech. rep., Rice University and Motorola Labs

Zhou, D., Chen, L., and He, Y. (2015). An Unsupervised Framework of Exploring Events on Twitter: Filtering, Extraction and Categorization. In *Proceedings of the Twenty-Ninth AAAI Conference on Artificial Intelligence* (Austin, Texas, USA: The AAAI Press), 2468–2474

Zhou, D., Chen, L., Zhang, X., and He, Y. (2017). Unsupervised Event Exploration from Social Text Streams. *Intelligent Data Analysis* 21, 849–866

Zhou, Y., De, S., and Moessner, K. (2016). Real World City Event Extraction from Twitter Data Streams. *Procedia Computer Science* 98, 443–448
